# Enviromics: understanding aging

**DOI:** 10.18632/aging.101709

**Published:** 2018-12-14

**Authors:** Theodore R. Smith, Aruni Bhatnagar

**Affiliations:** 1 The Envirome Institute, University of Louisville, KY 40292, USA; 2 Department of Pharmacology and Toxicology, University of Louisville, KY 40292, USA; 3 Department of Medicine, University of Louisville, KY 40292, USA

**Keywords:** envirome, cardiovascular disease, genome, lifespan

Human aging is determined by interactions between genetic and environmental factors. Although variations in the rate of aging across species suggests a strong role of genetics, heritability of lifespan observed within each species is < 35% [1], indicates that the environment plays an predominant role in aging. The reliability theory of aging portrays organisms as mechanical systems that contain components with varying probabilities of failure [2]. Complex organisms have redundancy in vital systems (perhaps better understood as the capacity to self-repair) so that every occurrence of damage does not result in death, but rather, the organism accumulates defects (due to inefficient repair) that ultimately exhaust reparative capacity. While in the context of this theory, the frequency and severity of damage has been thought to be determined, at least in part, by the environment, there are few, if any, conceptual models with robust explanatory power and predictive capacity to account for the influence of the environment on the rate of aging. In this regard, the concept of the envirome, analogous to the genome, could provide a useful ontological model for studying the relationship between the environmental circumstance and genetic predisposition [3,4].

Broadly, the envirome could be thought of as an integrated set of natural, social, and personal environmental domains. The natural domain of the environment consists of ecological and geographic conditions, whereas the social environment, which lies within the natural environment, includes the built environment, social networks, and culture. Lastly, the personal environment lies within the social environment and includes the factors specific to an individual [3,4]. In this model, interactions of the natural and social domains of the envirome with the genome could be viewed as the major determinants of aging. Aging, in turn, could be viewed as a progressive accrual of damage or unrepairable injury that results from a mismatch between the envirome and the genome and from exposure to adverse environmental conditions such as low socioeconomic status, smoking or air pollution.

**Figure 1:**
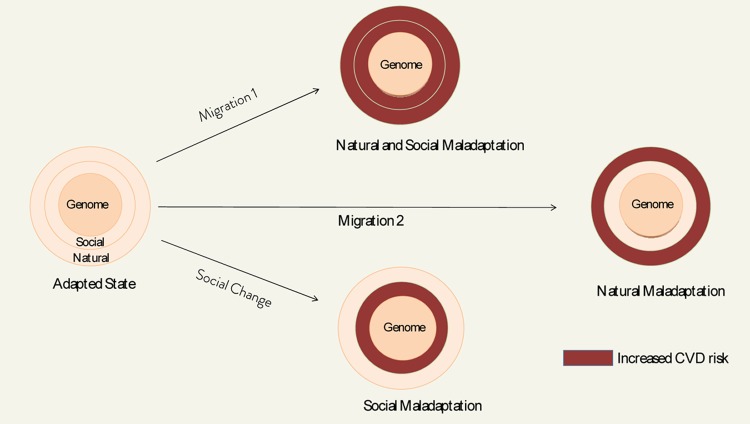
Simplified model of genome-envirome risk modification

Under ideal conditions, the genome could be viewed as adapted or matched with the natural and social domains of the envirome (See Figure 1). This state may be approximated in populations with long ancestry of living under relatively stable environmental conditions, e.g., Tibetans living on the Tibetan plateau, who seem to be adapted to their natural environment. However, when humans migrate to another culture in a new natural environment (Migration 1), their genomes are no longer adapted to the envirome. This mismatch compromises fitness and accelerates aging. Such a mismatch between ancestral genomes and contemporaneous enviromes could in principle account for the higher risk of cardiovascular disease in Japanese who migrate to the US, or accelerated aging in African-American, who age faster than US Whites (natural and social maladaptation). A mismatch between the genome and the envirome can also occur when there is a large change in the social environment, without large changes in the natural environment, e.g., Westernization of China led to a 50% increase in CVD mortality in men between 1984-1999 (social maladaptation) [4]. There are also instances of natural mismatch, i.e., when populations migrate from one natural environment to another (Migration 2), without a significant change in culture, e.g., Han Chinese migrating to Tibet, who experience chronic mountain sickness due to moving in a different (high altitude) environment [5] (natural maladaptation).

In the long run, living in new enviromes can result in evolutionary selection of adaptive alleles, which can restore the adaptive state. However, this change takes many generations, over which the human social environment changes yet again, leading to a persistent genome-envirome mismatch. Thus, human populations, due to constant migration and social development remain in a state of constant genome-envirome mismatch and exposure to adverse environmental factors. In terms of the reliability theory, the preexisting mismatch could be viewed as a set of pre-existing defects that may exist not only in early life, but *in utero* as well. The presence of initial defects, due to *a priori* genome-envirome mismatch, distinguishes humans from mechanical systems (in which all elements are functional from the outset) and is compatible with the exponential increase in failure rate with age (Gompertz law) observed in all human populations. From this it follows that even small improvements in the genome-envirome match that minimize *a priori* defects can potentially result in large gains in lifespan. In other words, enviromic fitness of parents, optimal ontogenesis, and the lack of adverse events in early life could lead to significant increases in lifespan. Additionally, modifications in the social and personal domains of the envirome that make them more concordant with genome or minimize adverse exposures, could deaccelerate aging, even later in life. Further work is required to assess the applicability of the enviromic model to aging research and to identify specific targets in the social and personal domains of the envirome that could be modified to minimize premature aging and increase human lifespan.
